# Changes in perceptions of antibiotic stewardship among neonatal intensive care unit providers over the course of a learning collaborative: a prospective, multisite, mixed-methods evaluation

**DOI:** 10.1038/s41372-023-01823-0

**Published:** 2023-11-24

**Authors:** Nabeel Qureshi, Jack Kroger, Kenneth M. Zangwill, Neha S. Joshi, Kurlen Payton, Peter Mendel

**Affiliations:** 1 RAND Corporation, Santa Monica, CA USA; 2 Pardee RAND Graduate School, Santa Monica, CA USA; 3 Division of Pediatric Infectious Diseases and The Lundquist Institute at Harbor-UCLA Medical Center, Torrance, CA USA; 4 Stanford University, Division of Pediatric Hospital Medicine, Palo Alto, CA USA; 5 Cedars-Sinai Medical Center, Department of Pediatrics, Division of Neonatology, Los Angeles, CA USA; 6 California Perinatal Quality Care Collaborative, Stanford, CA USA

**Keywords:** Paediatrics, Antimicrobial therapy

## Abstract

**Objective:**

To assess clinician perceptions towards the value and implementation of antibiotic stewardship (AS) in neonatal intensive care units (NICU).

**Study design:**

We performed a mixed-methods study of AS perceptions (prescribing appropriateness, importance, activity, capacity) using surveys and interviews in 30 California NICUs before and after a multicenter collaborative (Optimizing Antibiotic Use in California NICUs [OASCN]).

**Results:**

Pre-OASCN, 24% of respondents felt there was “a lot of” or “some” inappropriate prescribing, often driven by fear of a bad outcome or reluctance to change existing practice. Clinicians reported statistically significant increases in AS importance (71 v 79%), perceived AS activity (67 v 87%), and more openness to change after OASCN (59 v 70%). We identified other concerns that lessen AS effort.

**Conclusion:**

OASCN increased perceived AS activity and openness to change in AS practices among NICU prescribers. Greater attention to subjective concerns should augment AS improvement.

## Introduction

Existing literature has documented wide variation in overall antibiotic utilization rates (AUR) in the NICU setting; indeed, rates of inappropriate use often far exceed that of proven infection [1]. This clinical reality is the source of much discussion in the literature and continues despite national entreaties to increase infrastructural support and individual effort toward improved antibiotic stewardship (AS). Updated NICU-specific clinical practice guidelines for early-onset sepsis for term babies (a significant source of inappropriate antibiotic use) were published in 2018 [2, 3], but the extent to which these guidelines have impacted antibiotic use is unclear. Clinical guidelines for antibiotic use are also not yet available for EOS in preterm babies or other conditions presenting in the NICU setting.

AS programs improve antibiotic use practices and patient outcomes in a wide variety of settings and patient populations [4, 5]. NICUs, however, have unique challenges with implementing stewardship practices, including provider- and institution-level barriers [6, 7]. Among these barriers are a lack of clinical data to support definitive treatment plans for many specific infectious syndromes and a lack of readily available pediatric-trained infectious disease doctors in many units outside of urban areas [8]. Previous studies have also highlighted the lack of access to formalized stewardship programs and activities targeted towards reduction in antibiotic use; as well, these studies typically reflect practice from large volume, high acuity NICUs.

Perceptions represent a key structural determinant of quality improvement (QI) as defined by the Donabedian Model [9]. Most studies of stewardship efforts in NICUs largely focus on education about AS or specifically defined clinical management choices that impact AUR, rather than perceptions towards AS and the capacity to change practice [10]. Previous studies in and out of the NICU [7, 11–13] have noted the importance of perceptions in quality improvement (QI) initiatives. Understanding provider perceptions can help gauge provider belief about whether there is a need for stewardship and assess buy-in to stewardship activities and their engagement with potential practice change, which have downstream impacts on participation and willingness to change during the QI effort.

While shifting perceptions, attitudes, and values of prescribers is clearly an important part of successful adoption of stewardship practices and sustained reductions in antibiotic use, the literature on this concept is limited [14, 15], and none are available for the NICU setting beyond a survey of trainees’ knowledge of and training preferences for AS [11]. As part of a multisite quality improvement AS collaborative to safely reduce antibiotic use amongst 30 participating NICUs, we examined changes in providers’ perceptions and attitudes towards initiation, implementation, and expectations of their NICU’s AS efforts, over time.

## Methods

### OASCN collaborative intervention

We conducted a mixed-methods study to evaluate longitudinal changes in the perceptions and experiences of participants in a large AS collaborative among California NICUs. The collaborative, called the Optimizing Antibiotic Stewardship in California NICUs (OASCN, www.cpqcc.org/improvement/projects/OASCN), was conducted in conjunction with the California Perinatal Quality Care Collaborative (CPQCC) in partnership with the RAND Corporation, Lundquist Institute at Harbor-UCLA Medical Center, and University of Southern California. CPQCC is a statewide network of >130 California NICUs that works to improve the quality of clinical care through collaborative data collection and quality improvement projects [16]. OASCN used a blended quality improvement (QI) collaborative [17] and Extension for Community Healthcare Outcomes (ECHO) tele-learning model [18] to scale up dissemination and implementation of AS in NICUs located across California. This blended design resulted in a multi-pronged collaborative intervention that targeted perceptual and behavioral change around antibiotic prescribing among providers through case-based guided learning, support for internal dissemination of collaborative learnings and implementation of process and infrastructural changes within participating NICUs, as well as ongoing trend feedback on outcomes.

The collaborative began with 31 NICU sites and hosted 60-min Learning Sessions via Zoom every two weeks from March 2021 through February 2022. All clinical staff in these NICUs (physicians, nurses, pharmacists, trainees) were invited to participate. The biweekly Learning Sessions typically included review of patient cases chosen and presented by participants, as well as a short didactic presented by expert panel members or guest experts on a stewardship topic of interest. Other Learning Session activities included feedback of monthly antibiotic use trends from data submitted by the participating NICUs and presentations by participants on stewardship improvement strategies, challenges, and progress. The OASCN collaborative also provided other resources for participants, including: a newsletter with summary of learning points distributed after each Learning Session; “Office Hours” sessions on special topics (e.g., QI methods, pharmacy, and blood culture best practices) between Learning Sessions; an email listserv; a QI Fundamentals online course (offered through CPQCC, with continued medical education [CME] credit); an online QI data submission and analytics portal; and a file sharing site with archived Learning Session slide decks, didactic videos, other session materials, and a curated reference library.

Sites voluntarily enrolled in OASCN and were actively encouraged to focus over the “intensive collaborative” intervention year on improving AS and changing antibiotic use practices. Participating sites designated a site leader to be administratively responsible for overseeing data reporting, encouraging staff to participate in the Learning Sessions and other OASCN activities, and disseminating information from the Learning Sessions more broadly among NICU staff. In many participating NICUs, the site leader also headed AS efforts and/or a stewardship team, while in different sites that role could be played by other clinical leaders and champions. Approximately 60% of the site leaders were neonatologists, 30% nurse leaders (i.e., nurse manager, clinical nurse specialist or nurse educator), and the rest neonatal nurse practitioners (NNP) or clinical pharmacists.

### Data collection

This paper utilizes two sources of data collected as a part of the OASCN evaluation. The first source is a survey that all prescribing clinicians (“prescribers”) in participating NICUs were invited to complete, which included questions on their perceptions of AS at their site and on a series of antibiotic prescribing clinical vignettes. The survey was administered at two timepoints: once before the start of OASCN and again at the end of the first year, which concluded the “intensive collaborative” intervention activities. The second data source came from a set of semi-structured qualitative interviews with site leaders during those same pre- and post-intervention time periods to obtain their perspectives on antibiotic prescribing practices and stewardship improvement experiences within their sites.

The data from the survey used in this analysis focuses on prescribers’ perceptions of AS at their site, specifically those questions related to the perceived (a) appropriateness of antibiotic use in their NICU; (b) importance of stewardship; (c) level of active ongoing stewardship, and (d) capacity or interest in initiating new stewardship efforts during the OASCN collaborative. Responses related to need for active stewardship were measured using a four-point Likert scale (e.g., “a lot of”, “some”, “a little”, or “very little” inappropriate prescribing) and those related to importance, ongoing effort, and interest were measured using a five-point Likert scale (“not at all”, “a little bit”, “somewhat”, “very”, or “extremely”). We calculated top-two box scores for each, calculating the percentage of participants that answered “a little” or “very little” on the four-point and “very” or “extremely” on the five-point scale.

The survey also collected data assessing respondents’ professional roles and their length of time in that role and separately in that NICU site. For comparison purposes, we utilized CPQCC administrative data to classify the size of each NICU relative to all other CPQCC sites (representing over 90% of NICUs in California) as measured by total annual NICU admissions in terciles (small = ≤229 admissions, medium = >229 to ≤365 admissions, and large = >365 admissions). The survey was fielded electronically using the SelectSurvey platform for 8 weeks before the start of the collaborative and for 10 weeks at the end of the collaborative. Non-respondents were sent weekly email reminders, and all were offered a $30 Amazon gift card upon completion.

The interviews were conducted by two evaluation team members (PM and NQ) with site leaders (including the formal site lead and up to two other clinical leaders) from 30 of the participating NICUs using a semi-structured format lasting approximately 45–60 min. The interviews included questions on the perceived appropriateness of antibiotic usage in their NICUs, resources available for QI and AS activities, challenges and progress with stewardship improvement, and (in the post-intervention interview) their site’s experiences in the OASCN collaborative.

This research was approved by the RAND Human Subjects Protection Committee, the Stanford Panel on Medical Human Subjects, and the John F. Wolf Human Subjects Committee of The Lundquist Institute, in accordance with the Declaration of Helsinki. All participants provided consent to participate in surveys (written consent) and interviews (oral consent). The datasets generated and analyzed during the current study are not publicly available due to other ongoing analyses and manuscript writing in progress using the same data but are available from the corresponding author on reasonable request.

### Data analysis

We assessed change between the pre- and post-OASCN survey responses using univariate and bivariate analyses considering those that completed both the pre- and the post-OASCN survey (longitudinal cohort) and all those that completed either of the surveys. We calculated mean differences and proportion of top-two box responses overall and disaggregated by provider role, time in their role, and time at their site. We also compared responses by site leaders and non-site leader prescribers, by previous experience in an AS collaborative, and by site tercile. For all comparisons, we report p-values from two-sided t-tests and Chi-squared tests and significance was defined as p ≤ 0.05. All underlying distributions were normal and had similar variances. All quantitative analyses were conducted in Stata MP17 [19].

We conducted content coding and thematic analysis of the semi-structured interview data [20] using the Dedoose online qualitative software platform [21]. Three coders reviewed the transcripts and applied codes using a preliminary codebook defined by topics included in an interview guide. Themes that emerged through the coding were discussed during weekly qualitative team meetings for possible inclusion in the codebook. The coding team co-coded three transcripts of the 30 pre-OASCN site leader interviews, the 1st, 15th, and 30th transcripts (10% of transcripts), to ensure consistency throughout the coding process [22]. Inconsistencies in coding were discussed and resolved by consensus. After completing all rounds of co-coding, we calculated inter-rater reliability test scores. After the first transcript, we calculated a kappa = 0.75 and after the final transcript, we calculated a kappa = 0.87, indicating very good agreement among coders throughout the coding process [23]. We used the same codebook on the post-OASCN interviews and followed a similar coding consistency approach.

## Results

### Participants

Pre-OASCN, we interviewed site leaders from 30 NICUs; 26 (87%) of whom also completed the post-OASCN interview. The pre-OASCN survey was sent to 349 eligible prescribers across the units, of whom 256 (73%) responded. In the post-OASCN survey, we received 194 (63%) responses among the 309 fielded surveys. A total of 297 individuals participated in either the pre- or post-OASCN survey. For any individual NICU, we received responses from at least 50% of associated prescribers at a given site in the pre-OASCN survey (between 2 and 24 prescribers per site) and 40% in the post-OASCN survey (between 2 and 17 prescribers per site). Table 1 describes the 297 individuals who completed either the pre- or post-OASCN survey, and those that completed both surveys i.e., the longitudinal cohort (*N* = 166, 65% of the cohort that took the pre-survey). The majority of participants were neonatologists, followed by neonatal fellows, hospitalists/pediatricians, and advanced practice providers (nurse practitioner or physician assistant). Additionally, the majority of respondents had at least 10 years of experience in their roles (excluding fellows), with a plurality spending most of that time at their current site. The longitudinal cohort was similar to the entire sampled cohorts in the distribution of these characteristics (Table 1).

**Table 1:** Sample characteristics of respondents to the OASCN prescriber survey (pre- and post-intervention).

	Pre-intervention survey *N* (%)	Post- intervention survey *N* (%)	Longitudinal cohort *N* (%)
Clinical role			
Neonatologist	152 (59.3%)	122 (62.9%)	111 (66.9%)
Neonatal fellow	34 (13.2%)	30 (15.5%)	21 (12.7%)
Hospitalist/pediatrician	39 (15.2%)	25 (12.9%)	20 (12.1%)
Nurse practitioner/physician assistant	31 (12.1%)	17 (8.8%)	14 (8.4%)
Time in role			
≤1 year	21 (8.2%)	7 (4.1%)	12 (7.2%)
>1 year and ≤5 years	65 (25.3%)	53 (27.3%)	40 (24.1%)
>5 years and ≤10 years	37 (14.4%)	30 (15.5%)	29 (17.5%)
>10 years and ≤15 years	49 (19.1%)	36 (18.6%)	33 (19.9%)
>15 years	80 (31.2%)	67 (34.5%)	52 (31.3%)
Time at site			
≤1 year	25 (9.8%)	10 (5.2%)	17 (10.2%)
>1 year and ≤5 years	89 (34.7%)	66 (34.0%)	55 (33.1%)
>5 years and ≤10 years	41 (16.0%)	35 (18.0%)	29 (17.5%)
>10 years and ≤15 years	39 (15.2%)	33 (17.0%)	28 (16.9%)
>15 years	58 (22.6%)	50 (25.8%)	37 (22.3%)
Total	256 (100%)	194 (100%)	166 (100%)

The longitudinal cohort consists of prescribers who responded to both the pre- and post-intervention survey.

### Perceptions of current antibiotic practice

Within the longitudinal cohort overall, >76% of prescribers characterized their units’ antibiotic use practice as having “little” or “very little” inappropriate prescribing, which did not vary significantly pre- versus post-OASCN (Table 2). Looking by collaborative role, site leaders at the pre-OASCN time point reported significantly higher perceived appropriate antibiotic use than other prescriber participants (92% v 73%, 3.62 v. 3.13, *p* = 0.01; Table 3), however this difference between groups was no longer significant by the post-OASCN survey. Perceptions of appropriate prescribing also did not vary significantly by participation in OASCN Learning Sessions (Table 4), nor by clinical role (neonatologists versus other prescribers), time in clinical role, or time at NICU site (Table 4, and Appendix Tables A.1, and A.2, respectively).

**Table 2:** Survey responses of the longitudinal cohort, pre- versus post-OASCN intervention.

Query	Number of responses	Pre-OASCN mean (% top-two box^a^)	Post-OASCN mean (% top-two box)
Current practice			
In general, how would you rate the clinical appropriateness of antibiotic prescribing by clinicians in this NICU?^b^	163	3.20 (76.1%)	3.27 (77.9%)
Importance			
How important do you view antibiotic stewardship to be, compared to other priorities in this NICU?	165	4.15 (84.9%)	4.24 (89.1%)
In general, how important do you think other clinicians in this NICU view antibiotic stewardship to be, compared to other priorities in the NICU?	164	3.84 (71.3%)	3.93 (78.7%)*
How would you rate the importance placed on antibiotic stewardship by this NICU’s leadership?	166	4.21 (81.9%)	4.28 (87.4%)
How would you rate the importance placed on antibiotic stewardship by this hospital’s leadership?	118	3.88 (73.7%)	3.97 (74.6%)
Activity			
How active has this NICU been in trying to improve antibiotic stewardship in the past year?	158	3.84 (67.1%)	4.32 (87.3%)***
How active has this hospital been in trying to improve antibiotic stewardship in the past year?	107	3.72 (70.1%)	3.91 (72.0%)
Capacity			
How would you rate the general openness of clinicians in this NICU to changing their antibiotic prescribing practice?	162	3.70 (59.3%)	3.89 (70.4%)***
How difficult do you think it would be for this NICU to safely reduce its antibiotic use?	162	2.22 (4.3%)	-

The longitudinal cohort consists of prescribers who responded to both the pre- and post-OASCN survey (*N* = 166).

^a^Percent top-two box defined as the percent who answered “a little” or “very little” on the four-point Current Practice Likert items and “very” or “extremely” on the five-point Importance, Activity, and Capacity items.

^b^Responses are in terms of inappropriate prescribing, so top-two box responses are “a little inappropriate prescribing” and “very little inappropriate prescribing”.

Pre-post differences: *p < 0.05, ***p < 0.001.

**Table 3:** Survey responses of the longitudinal cohort, by collaborative role (site leaders versus other prescriber participants).

Survey question	Number of responses	Type of collaborative role	Pre-OASCN mean (% top-two box^a^)	Post-OASCN mean (% top-two box)
Current practice				
In general, how would you rate the clinical appropriateness of antibiotic prescribing by clinicians in this NICU?^b^	24	Site leaders	3.62 (91.7%)	3.50 (83.3%)
	139	Other participants	3.13 (73.4%)^	3.23 (77.0%)
Importance				
How important do you view antibiotic stewardship to be, compared to other priorities in this NICU?	24	Site leaders	4.12 (83.3%)	4.25 (91.7%)
	141	Other participants	4.15 (85.1%)	4.24 (88.7%)
In general, how important do you think other clinicians in this NICU view antibiotic stewardship to be, compared to other priorities in the NICU?	24	Site leaders	3.91 (70.8%)	3.91 (83.3%)
	140	Other participants	3.83 (71.4%)	3.91 (83.3%)
How would you rate the importance placed on antibiotic stewardship by this NICU’s leadership?	24	Site leaders	4.33 (83.3%)	4.37 (95.8%)
	142	Other participants	4.19 (81.7%)	4.26 (85.9%)
How would you rate the importance placed on antibiotic stewardship by this hospital’s leadership?	21	Site leaders	3.90 (76.2%)	3.95 (76.2%)
	97	Other participants	3.87 (73.2%)	3.97 (74.2%)
Activity				
How active has this NICU been in trying to improve antibiotic stewardship in the past year?	24	Site leaders	3.83 (66.7%)	4.33 (91.7%)*
	134	Other participants	3.84 (67.2%)	4.32 (86.6%)**
How active has this hospital been in trying to improve antibiotic stewardship in the past year?	18	Site leaders	3.77 (77.8%)	3.77 (72.2%)
	89	Other participants	3.70 (68.5%)	3.94 (71.9%)*
Capacity				
How would you rate the general openness of clinicians in this NICU to changing their antibiotic prescribing practice?	23	Site leaders	3.91 (69.6%)	3.82 (69.6%)
	139	Other participants	3.66 (57.6%)	3.90 (70.5%)***
How difficult do you think it would be for this NICU to safely reduce its antibiotic use?	24	Site leaders	2.12 (3.6%)	-
	142	Other participants	2.34 (8.3%)	-

The longitudinal cohort consists of prescribers who responded to both the pre- and post-OASCN survey (*N* = 166).

^a^Percent top-two box defined as the percent who answered “a little” or “very little” on the four-point Current Practice Likert items and “very” or “extremely” on the five-point Importance, Activity, and Capacity items.

^b^Responses are in terms of inappropriate prescribing, so top-two box responses are “a little inappropriate prescribing” and “very little inappropriate prescribing”.

Pre-post differences: *p < 0.05, **p < 0.01, ***p < 0.001.

Pre-collaborative differences by type: ^*p* < 0.05.

**Table 4:** Survey responses of longitudinal cohort, by participation in OASCN learning sessions (participated versus did not participate).

Query	Number of responses	Learning session participation^a^	Pre-OASCN mean (% top-two box^b^)	Post-OASCN mean (% top-two box)
Current practice				
In general, how would you rate the clinical appropriateness of antibiotic prescribing by clinicians in this NICU?^c^	63	Participated	3.62 (91.7%)	3.50 (83.3%)
	100	Did not participate	3.13 (73.4%)	3.23 (77.0%)
Importance				
How important do you view antibiotic stewardship to be, compared to other priorities in this NICU?	64	Participated	4.23 (85.9%)	4.39 (92.2%)
	101	Did not participate	4.10 (84.2%)	4.15 (87.1%)+
In general, how important do you think other clinicians in this NICU view antibiotic stewardship to be, compared to other priorities in the NICU?	64	Participated	3.84 (67.2%)	3.97 (79.7%)
	100	Did not participate	3.85 (74.0%)	3.97 (79.7%)
How would you rate the importance placed on antibiotic stewardship by this NICU’s leadership?	64	Participated	4.27 (84.3%)	4.41 (93.8%)
	102	Did not participate	4.18 (80.4%)	4.21 (83.3%)
How would you rate the importance placed on antibiotic stewardship by this hospital’s leadership?	49	Participated	3.94 (77.6%)	3.94 (77.6%)
	69	Did not participate	3.84 (71.0%)	4.00 (72.5%)
Activity				
How active has this NICU been in trying to improve antibiotic stewardship in the past year?	62	Participated	3.69 (54.8%)	4.40 (90.3%)***
	96	Did not participate	3.94 (75.0%)	4.28 (85.4%)***
How active has this hospital been in trying to improve antibiotic stewardship in the past year?	43	Participated	3.58 (62.8%)	3.79 (67.4%)
	64	Did not participate	3.81 (75.0%)	4.00 (75.0%)
Capacity				
How would you rate the general openness of clinicians in this NICU to changing their antibiotic prescribing practice?	63	Participated	3.71 (58.7%)	3.92 (71.4%)*
	99	Did not participate	3.70 (59.6%)	3.88 (69.7%)*
How difficult do you think it would be for this NICU to safely reduce its antibiotic use?	64	Participated	2.22 (7.8%)	-
	102	Did not participate	2.37 (2.0%)	-

The longitudinal cohort consists of prescribers who responded to both the pre- and post-OASCN survey (*N* = 166).

^a^Participated includes prescribers who participated in at least one OASCN Learning Session.

^b^Percent top-two box defined as the percent who answered “a little” or “very little” on the four-point Current Practice Likert items and “very” or “extremely” on the five-point Importance, Activity, and Capacity items.

^c^Responses are in terms of inappropriate prescribing, so top-two box responses are “a little inappropriate prescribing” and “very little inappropriate prescribing”.

Pre-post differences: *p < 0.05, **p < 0.01, ***p < 0.001.

Post-collaborative difference by type: ^+^*p* < 0.05.

In qualitative interviews, site leaders were asked to assess the general appropriateness of antibiotic use in their NICUs before the OASCN Collaborative. Overall, 16/30 (53%) reported having overall appropriate antibiotic prescribing practices, 9 (30%) having overall inappropriate practice overall, and 5 (17%) discussing areas that were appropriate and other areas that were inappropriate. We also queried appropriateness within specific areas of AS (i.e., drug choice, starting or stopping antibiotics, and drug choice and duration). No site leaders reported issues with appropriateness of drug or dose. However, among the 14 site leaders who reported having some inappropriate prescribing practices, 9 (30% overall, 64% of those reporting inappropriate prescribing) noted inappropriate practices around starting antibiotics, and 10 (33% overall, 71% of those reporting inappropriate prescribing) reported inappropriate practices around stopping antibiotics. Major drivers associated with starting antibiotics included fear of a bad outcome, empiric use prior to culture results becoming available, and in some, infants already being on antibiotics before admission to the NICU, and provider reluctance to change long-standing practices:

*“I don’t know what it’s going to take to do that, but there are certain concerns that infection in babies is a rare event, and so they’re very worried about missing that one baby [who] should have been on antibiotics and wasn’t.” – Urban setting NICU in Southern California*

*“So now that we’re taking care of the smaller, younger babies, like I said, I already can tell there’s some kneejerk reactions to putting her on antibiotics, when maybe she didn’t need that, at least for like… 48* *hours and see how she does type thing, which is like a very common NICU practice.” – Urban setting NICU in Southern California*

### Perceptions of AS importance

In the longitudinal cohort, prescribers reported high levels of importance of AS to self, lower levels of importance from other clinicians, higher levels from their NICU leadership, and lower levels from hospital leaders, though all differences from pre- to post-OASCN surveys were not statistically significant except for those of other (non-neonatologist) clinicians (Table 2). Of note, many prescribers (48/166, 29%) reported not knowing how their hospital leadership perceived the importance of AS. There were also few significant differences by provider type, though neonatologists generally viewed AS as more important and active in their units compared to others (Appendix Table A.3). As noted in Table A.1, post-OASCN, those who had spent ≥10 years in their role reported significantly higher levels of AS importance among themselves, other clinicians, NICU leadership, and hospital leadership, compared to those with less experience.

Site leaders reported being highly motivated to participate in the collaborative and improve AS. When asked about other resources for AS, most site leaders did not think there was a strong connection to hospital leadership or hospital-wide AS efforts.

*“I don’t think we’re the only one [NICU] where a NICU in a very large, adult-focused hospital, we’re kind of tucked away in a corner a lot of the times, and as long as our metrics are good then they just support us with administrative type of resources, for example, maintaining a team of nurses to look at data.” – Urban setting NICU in Northern California*

*“[In the NICU,] we tend to be kind of isolated. We’re like our own functioning, tiny, mini hospital.” – Urban setting NICU in Northern California*

NICUs may be perceived as a different entity and there may be a reluctance to engage fully with NICUs on wider hospital efforts. Site leaders noted that even though they function separately from the hospital, hospital leadership is still supportive of participation in AS efforts.

*“…Administration and everyone else… they don’t know NICU, they don’t know NICU care, and it kind of worries them and scares them so they usually stay out and they don’t— there’s really not a lot of support for it.” – Urban setting NICU in Southern California*

One pharmacist involved in AS noted that the pharmacy needs of the NICU are also distinct from the wider hospital. Given the scope of antibiotic needs throughout the hospital, individuals involved in stewardship focus on the adult side and leave the NICU to work on its own.

*“…because [antibiotic stewardship responsibility is on] one person for the whole hospital, I focus mainly on adults because that’s where my training is. And NICU is always just kind of run independently” – Urban setting NICU in Southern California*

### Perceptions of AS activity

Prescribers reported “very” or “extremely” high levels of stewardship activity in the pre- and post-OASCN periods, and with a statistically significant increase over time (67% v. 87%, 3.84 v 4.32, *p* < 0.001), similar to site leaders alone (Table 3). This increase was more pronounced among those who participated in at least one Learning Session (Table 4) or had ≥10 years in their role (Appendix Table A.1). The overall pre-post trends by provider type were similar to the overall trends, but neonatologists reported significantly larger activity levels in the post-collaborative survey than those with other roles (92% v 79%, 4.45 v. 4.07, *p* = 0.003; Appendix, Table A.3).

The pre- to post-OASCN increase in AS activity is larger and significant for small sized (68% v 89%, 3.89 v. 4.34, *p* = 0.048; Appendix, Table A.4) and medium-sized (69% v 93%, 3.91 v. 4.44, *p* < 0.001) NICUs, but not for the larger NICUs. There was also a significant difference in perceived hospital-wide activity reported by those with ≥10 years in role (3.69 v. 4.05, p < 0.001; Appendix Table A.1), although many (59/166, 36%) reported not knowing the level of activity related to AS in the hospital. Other prescribers, but not site leaders, also reported an increased perception of hospital-wide AS activity during OASCN (3.94 v. 3.70, *p* = 0.03; Table 3).

In qualitative interviews, over the course of the collaborative, all site leaders reported improvements to activity to improve AS. Site leaders revealed several activities related to AS that were implemented in the NICU during the collaborative, including updating antibiotic use guidelines (18/30, 60%); implementing more standardized rule out sepsis protocols (9/30, 30%), hard antibiotic stops at 48 and 36-h (8/30, 27%), antibiotic timeouts (2/30, 7%); and adding sepsis calculators into their electronic medical record (2/30, 7%). Sites implemented between one and six changes to improve AS, with sites implementing a median of 4 changes. Site leaders reported that clinicians did make progress towards more consistently appropriate prescribing practices over the course of the collaborative as a consequence of these implemented activities.

### Perceptions of AS capacity

Prescribers were asked two items in the pre-OASCN survey about the capacity to reduce antibiotic use (openness to and difficulty in reducing antibiotics), and one item in the post-collaborative survey (openness to reducing antibiotics). Prescribers reported high levels of openness in the pre- and post-OASCN surveys and a statistically significant improvement over time (59% pre-OASCN as “very” or extremely” open versus 70% post-OASCN; Table 2). In the pre-OASCN survey, almost all prescribers reported that it would be a little to somewhat difficult to reduce antibiotic use safely (4.3% reported very or extremely difficult to reducing antibiotic use safely).

In the pre-OASCN survey, neonatologists reported significantly higher levels of openness to changing practice than those in other roles (3.85 v. 3.41, *p* = 0.007; Appendix Table A.3), though this gap shrank in the post-survey despite remaining significantly different. It was also moderated by time in role and site, with those with ≥10 years in their role reporting increased levels of openness in the post-OASCN survey, relative to more junior peers who did not report a significant increase. This was driven by the larger NICUs (Table A.4). Respondents that participated in at least one Learning Session had greater gains in their perception of openness to change during OASCN than those that did not participate (Table 4).

Key barriers to implementing AS practices reported by site leaders included difficulty engaging providers and getting their buy-in. Among the most cited concerns were fear of a bad clinical outcome from withholding antibiotics, stopping antibiotics too early (particularly for preterm babies), the interest of maintaining existing long-standing practice (that often use more antibiotics than is recommended), and the desire to treat culture-negative sepsis and early-onset sepsis.

*“The biggest barrier is what we’ve done for 20 or 30 years. We have some older physicians in our practice who have practiced a certain way and given antibiotics at certain times for a really long time.” – Urban setting NICU in Northern California*

A consequence of participating in the OASCN collaborative, however, was once there was some buy-in to reduce their antibiotic use, providers did not observe a rise in adverse outcomes and were therefore more open to continued decreases.

*“But once we got the buy-in and they saw that there were no adverse outcomes in these babies that weren’t started on antibiotics, I think that that helped a lot. It helped be more smooth for the rest of the year.” – Urban setting NICU in Southern California*

We performed analyses on certain subgroups. In 2016–17, 10 (33%) of the OASCN sites participated in a less intensive AS collaborative [24]. Analyses of this group, in comparison to the entire OASCN cohort, did not reveal substantial differences in the perceptions of prescribing appropriateness, importance of AS, AS activity, or capacity. Prior collaborative participants did, however, report a higher perceived level of appropriate prescribing practices before the OASCN collaborative which was no longer significant post-OASCN (data not shown). Lastly, considering the entire cohort of 297 participants (compared to the longitudinal cohort), there were no statistically significant pre-to post-OASCN increases in responses for any question (Appendix Table A.5).

## Discussion

We assessed how a blended QI collaborative and ECHO tele-learning intervention—the OASCN collaborative—affected provider perceptions about antibiotic stewardship in the NICU setting. Such data as collected by this study have not been previously available and include information from a relatively large sample of neonatologists and other prescribers across 30 NICUs in California. Our mixed-methods approach identified favorable changes resulting from OASCN, particularly regarding prescribers’ perceptions of the level of clinician activity towards improving stewardship and the openness of providers to change prescribing practices. The general characteristics of our sample are similar to that described from all NICUs in California and consistent with other US studies of NICU clinician cohorts [25, 26], enhancing the generalizability of our findings.

It is also important to note that provider perceptions at the start of any AS effort can impact the likelihood of success towards encouraging provider buy-in and targeting of potential areas for such improvement activities [7, 11, 12]. Our data revealed encouraging changes in perceptions towards AS activities and expectations over time. A lack of such a shift might have suggested need for significant modifications to the overall QI approach and/or methods to engage clinicians.

Prescribers noted a significant increase in AS activities during the intervention, particularly in the small and medium-sized units. This improvement was even noted among prescribers who did not participate in the Learning Sessions, which suggests successful local dissemination of AS best practices and/or via other channels provided in a continuous way by the OASCN infrastructure (e.g. email newsletters distributed after each session to all participating site teams that summarized learning points, an email listerve, and an online resource repository of learning session didactic videos and slides, references, and other information) [27]. Thus, a multimodal approach utilizing multiple dissemination strategies within the unit level may be indicated to effectively increase awareness and knowledge of AS best practices within NICUs.

Yet, respondents overall had not perceived a change in the appropriateness of antibiotic prescribing by the end of the first year of the collaborative. Greater than 75% of prescribers believed that antibiotic use was generally appropriate prior to OASCN with little perceived change noted in the post-survey. This may reflect the time needed for increased activity to result in improved antibiotic stewardship, as well as for improved prescribing appropriateness (if achieved) to be perceived more widely among clinicians. Strategies to publicize data on improved prescribing practice and antibiotic use among NICU staff may reduce such lags in perceptions between activity and impact.

Despite not noting a significant movement in prescribing behavior within their NICUs, prescribers reported an increase in openness to practice change and specific activities implemented to accomplish it. The former was particularly true among the neonatologists and those in practice >10 years, the latter being previously shown to be more resistant to AS activity goals [28, 29]. Greater openness to practice change may be reflective of changes in group norms and “cognitive participation” that is associated with collective action and sustained behavior change [30], such as moving away from culture-negative sepsis [31].

In this milieu of openness to change, our interviews nonetheless revealed barriers to improved stewardship related to very personal concerns among providers such as fear of a bad clinical outcome from not using antibiotics, stopping antibiotics too early, fear of changing existing long-standing local practices, and the pressure to treat culture-negative sepsis and some presentation associated with early-onset sepsis. In addition, we found a general lack of understanding of NICU prescribers on hospital-wide attention to stewardship, and a reciprocal lack of attention to stewardship in the NICU by hospital AS programs during out interviews. Most published reviews highlight the more logistical barriers to implementing antibiotic stewardship in NICUs [6], not perception (e.g., limited or no neonatal data to support specific treatments, lack of infectious disease or neonatal pharmacy expert consultation). Individual-level concerns such as we identified have been shown in non-NICU settings to play a very important role if successful stewardship is to be achieved [32]. The particular vulnerability of babies requiring intensive care may make the more subjective and/or personal challenges to AS implementation perhaps even more challenging and should be a clear focus of AS programs in the NICU setting.

Our qualitative data identified specific target areas that could benefit from AS efforts, including education and discussion about when to start, stop, and for how long antibiotics should be given rather than on choice of antibiotic type or dose. This finding reflects the reality that prescribers often do not adhere to guidelines in a consistent fashion [33–35]; AS efforts should continue to focus on these core areas of decision making [6, 36, 37]. In our cohort, sites implemented a range of activities to improve prescribing practices, chief among those being implementing treatment guidelines and use of antibiotic timeouts and hard stops, which have been shown to reduce the use of antibiotics [37–39].

We found that certain incongruities existed between reported perceptions and actual practice. For example, we found that >95% of clinicians with ≥10 years of experience in their role or at their site reported AS as very or extremely important, contrary to the perception from site leaders that such providers may “*[practice] a certain way*” precluding an easy adoption of best practices. This divide is also reflected in the commonly reported belief that although our NICUs were typically in support of and active in stewardship. They also felt as if they functioned separately from the hospital, with individuals likening it to having a “small, mini-hospital” in the larger institution that does not acknowledge NICU AS effort and needs. Greater involvement of NICU staff in hospital-wide stewardship goals, policy development, and resource allocation may improve stewardship awareness and successful outcomes, as has been shown in other inpatient unit types [40].

This study has several limitations. First, our use of Likert scales to measure perceptions is limited by “top loading” of responses (i.e., individuals’ tendency to select extreme values), potentially restricting our ability to differentiate perceptions over time. In addition, the statistical testing results for Likert comparisons may not align as clinically relevant, a known limitation of this measurement approach [41]. We do, however, measure perceptions around several aspects of stewardship (importance, activity, capacity, appropriateness) and relate numerical changes with qualitative interview data to provide more inclusive assessments. Additionally, this study does not comprehensively associate perceptual changes with actual changes in AS; these effects will be examined in future analyses the OASCN evaluation is conducting on site-level changes in stewardship activities and antibiotic use outcomes over the course of the collaborative intervention. Finally, while the longitudinal cohort of providers (responding to both the pre- and post-OASCN survey and on which the analyses were based) included more than 40% of prescribers in the participating NICUs, those who did not respond to one or both survey timepoints may have had different attitudes towards antibiotic stewardship.

We believe our findings regarding perception of AS in the NICU setting identify potential areas of further interrogation such as a focus on the more subjective barriers to successful AS to augment and amplify the standard targets of guideline adherence, drug management, and other logistical processes. That we found greater perception of openness to change in larger NICUs and more perceived actual AS activity in small to medium-sized NICUs as a result of our OASCN collaborative intervention may point to dynamics heretofore not studied in NICUs.

### Supplementary information

Appendix

## Data Availability

The datasets generated and analyzed during the current study are not publicly available due to other ongoing analyses and manuscript writing in progress using the same data but are available from the corresponding author on reasonable request.
