# Supplementary material for: Changes in perceptions of antibiotic stewardship among neonatal intensive care unit providers over the course of a learning collaborative: a prospective, multisite, mixed-methods evaluation
Source: J Perinatol. 2023 Nov 24;44(1):62–70. doi: 10.1038/s41372-023-01823-0 (PMC10783543; doi:10.1038/s41372-023-01823-0)
Supplement: Supplementary file 1 — Appendix [file 41372_2023_1823_MOESM1_ESM.docx]

**Appendix:**

**Table A.1:** Survey responses of longitudinal cohort, by time in clinical role (>= 10 years versus <10 years)

| Time in Role | **Number of responses** | **Time in clinical role** | **Pre-OASCN mean  (% top-two box^a^)** | **Post-OASCN mean  (%top-two box)** |
| --- | --- | --- | --- | --- |
| **Current practice** | | | | |
| In general, how would you rate the clinical appropriateness of antibiotic prescribing by clinicians in this NICU^b^? | 84 | >= 10 Years | 3.26 (78.7%) | 3.28 (75.5%) |
|  | 79 | < 10 Years | 3.15 (72.1%) | 3.25 (77.2%) |
| **Importance** | | | | |
| How important do you view antibiotic stewardship to be, compared to other priorities in this NICU? | 84 | >= 10 Years | 4.25 (89.2%) | **4.36 (91.6%)** |
|  | 81 | < 10 Years | 4.04 (80.2%) | **4.11 (86.4%)+** |
| In general, how important do you think **other clinicians** in this NICU view antibiotic stewardship to be, compared to other priorities in the NICU? | 83 | >= 10 Years | 3.95 (78.3%) | **4.08 (83.1%)** |
|  | 81 | < 10 Years | 3.74 (64.2%) | **3.79 (74.0%)++** |
| How would you rate the importance placed on antibiotic stewardship by this **NICU’s leadership**? | 85 | >= 10 Years | 4.29 (82.3%) | **4.43 (88.2%)** |
|  | 81 | < 10 Years | 4.12 (81.4%) | **4.12 (86.4%)++** |
| How would you rate the importance placed on antibiotic stewardship by this **hospital’s leadership**? | 61 | >= 10 Years | **3.90 (73.7%)** | **4.14 (80.3%)**** |
|  | 57 | < 10 Years | 3.86 (73.6%) | **3.78 (68.4%)+** |
| **Activity** | | | | |
| How active has this **NICU** been in trying to improve antibiotic stewardship in the past year? | 82 | >= 10 Years | **3.86 (70.7%)** | **4.41 (89.0%)***** |
|  | 76 | < 10 Years | **3.81 (63.1%)** | **4.23 (85.5%)**** |
| How active has this **hospital** been in trying to improve antibiotic stewardship in the past year? | 58 | >= 10 Years | **3.69 (68.9%)** | **4.05 (75.8%)*** |
|  | 49 | < 10 Years | 3.75 (71.4%) | **3.75 (67.3%)+** |
| **Capacity** | | | | |
| How would you rate the general openness of clinicians in this NICU to changing their antibiotic prescribing practice? | 82 | >= 10 Years | **3.81 (67.0%)** | **4.07 (80.4%)**** |
|  | 80 | < 10 Years | 3.58 (51.2%) | **3.71 (60.0%)++** |
| How difficult do you think it would be for this NICU to safely reduce its antibiotic use? | 85 | >= 10 Years | **2.11 (3.57%)** |  |
|  | 81 | < 10 Years | **2.51 (5.13%)^** |  |
| **Note**: The longitudinal cohort consists of prescribers who responded to both the pre- and post-OASCN survey (N=166)  ^a^ Percent top-two box defined as the percent who answered “a little” or “very little” on the four-point Current Practice Likert items and “very” or “extremely” on the five-point Importance, Activity, and Capacity items  ^b^ Responses are in terms of inappropriate prescribing, so top-two box responses are “a little inappropriate prescribing” and “very little inappropriate prescribing  Pre-post differences: * p<0.05, ** p<0.01, *** p<0.001;  Pre-collaborative differences by type: ^ p<0.05, ^^ p<0.01, ^^^ p<0.001;  Post-collaborative differences by type: + p<0.05, ++ p<0.01, +++ p<0.001 | | | | |

**Table A.2:** Survey responses of longitudinal cohort, by time at NICU site (>= 10 years versus <10 years)

| Time at Site | **Number of responses** | **Time at NICU site** | **Pre-OASCN mean  (% top-two box^a^)** | **Post-OASCN mean  (%top-two box)** | |
| --- | --- | --- | --- | --- | --- |
| **Current practice** | | | | | |
| In general, how would you rate the clinical appropriateness of antibiotic prescribing by clinicians in this NICU^b^? | 65 | >= 10 Years | 3.23 (75.3%) | 3.26 (76.9%) | |
|  | 98 | < 10 Years | 3.19 (76.5%) | 3.27 (78.5%) | |
| **Importance** | | | | | |
| How important do you view antibiotic stewardship to be, compared to other priorities in this NICU? | 65 | >= 10 Years | **4.43 (95.3%)** | **4.41 (95.3%)** | |
|  | 100 | < 10 Years | **3.97 (78.0%)^^^** | **4.13 (85.0%)+++** | |
| In general, how important do you think **other clinicians** in this NICU view antibiotic stewardship to be, compared to other priorities in the NICU? | 64 | >= 10 Years | 4.09 (85.9%) | 4.15 (89.0%) | |
|  | 100 | < 10 Years | **3.69 (62.0%)^^^** | **3.80 (72.0%)+++** | |
| How would you rate the importance placed on antibiotic stewardship by this **NICU’s leadership**? | 65 | >= 10 Years | **4.44 (89.2%)** | **4.52 (92.3%)** | |
|  | 101 | < 10 Years | **4.05 (77.2%)^^^** | **4.12 (84.1%)+++** | |
| How would you rate the importance placed on antibiotic stewardship by this **hospital’s leadership**? | 46 | >= 10 Years | **3.91 (76.0%)** | **4.17 (82.6%)***** | |
|  | 72 | < 10 Years | 3.86 (72.2%) | 3.84 (69.4%) | |
| **Activity** | | | | | |
| How active has this **NICU** been in trying to improve antibiotic stewardship in the past year? | 64 | >= 10 Years | **4.01 (76.5%)** | **4.48 (90.6%)***** | |
|  | 94 | < 10 Years | **3.72 (60.6%)** | **4.22 (85.1%)***^** | |
| How active has this **hospital** been in trying to improve antibiotic stewardship in the past year? | 43 | >= 10 Years | 3.67 (69.7%) | 4.07 (76.7%) | |
|  | 64 | < 10 Years | 3.75 (70.3%) | 3.81 (68.7%) | |
| **Capacity** | | | | | |
| How would you rate the general openness of clinicians in this NICU to changing their antibiotic prescribing practice? | 64 | >= 10 Years | **3.95 (73.4%)** | **4.09 (82.8%)** | |
|  | 98 | < 10 Years | **3.54 (50.0%)^** | **3.76 (62.2%)*+++** | |
| How difficult do you think it would be for this NICU to safely reduce its antibiotic use? | 65 | >= 10 Years | **2.00 (1.54%)** |  | |
|  | 101 | < 10 Years | **2.51 (6.19%)^^** |  | |
| **Note**: The longitudinal cohort consists of prescribers who responded to both the pre- and post-OASCN survey (N=166)  ^a^ Percent top-two box defined as the percent who answered “a little” or “very little” on the four-point Current Practice Likert items and “very” or “extremely” on the five-point Importance, Activity, and Capacity items  ^b^ Responses are in terms of inappropriate prescribing, so top-two box responses are “a little inappropriate prescribing” and “very little inappropriate prescribing  Pre-post differences: * p<0.05, ** p<0.01, *** p<0.001;  Pre-collaborative differences by type: ^ p<0.05, ^^ p<0.01, ^^^ p<0.001;  Post-collaborative differences by type: + p<0.05, ++ p<0.01, +++ p<0.001 | | | | |  |

**Table A.3**: Survey responses of longitudinal cohort, by clinical role (neonatologists versus other prescribers)

|  | **Number of responses** | **Type of clinical role** | **Pre-OASCN mean  (% top-two box^a^)** | **Post-OASCN mean  (%top-two box)** |
| --- | --- | --- | --- | --- |
| **Current practice** | | | | |
| In general, how would you rate the clinical appropriateness of antibiotic prescribing by clinicians in this NICU^b^? | 108 | Neonatologists | 3.16 (72.1%) | 3.27 (76.2%) |
|  | 55 | Other prescribers | 3.29 (81.8%) | 3.25 (80.0%) |
| **Importance** | | | | |
| How important do you view antibiotic stewardship to be, compared to other priorities in this NICU? | 110 | Neonatologists | 4.19 (87.2%) | 4.29 (90.9%) |
|  | 55 | Other prescribers | 4.07 (80.0%) | 4.14 (85.4%) |
| In general, how important do you think **other clinicians** in this NICU view antibiotic stewardship to be, compared to other priorities in the NICU? | 109 | Neonatologist | 3.89 (73.3%) | 3.97 (79.8%) |
|  | 55 | Other prescribers | 3.76 (67.2%) | 3.87 (76.3%)+ |
| How would you rate the importance placed on antibiotic stewardship by this **NICU’s leadership**? | 111 | Neonatologists | 4.26 (84.6%) | **4.37 (90.0%)** |
|  | 55 | Other prescribers | 4.10 (76.3%) | **4.09 (81.8%)++** |
| How would you rate the importance placed on antibiotic stewardship by this **hospital’s leadership**? | 85 | Neonatologists | 3.87 (76.4%) | 4.00 (76.4%) |
|  | 33 | Other prescribers | 3.90 (66.6%) | 3.90 (69.7%) |
| **Activity** | | | | |
| How active has this **NICU** been in trying to improve antibiotic stewardship in the past year? | 106 | Neonatologists | **3.89 (66.9%)** | **4.45 (91.5%)***** |
|  | 52 | Other prescribers | **3.73 (67.3%)** | **4.07 (78.8%)*++** |
| How active has this **hospital** been in trying to improve antibiotic stewardship in the past year? | 76 | Neonatologists | 3.73 (68.4%) | 3.90 (72.3%) |
|  | 31 | Other prescribers | 3.67 (74.1%) | 3.93 (70.9%) |
| **Capacity** | | | | |
| How would you rate the general openness of clinicians in this NICU to changing their antibiotic prescribing practice? | 107 | Neonatologists | **3.85 (67.2%)** | **3.98 (73.8%)** |
|  | 55 | Other prescribers | **3.41 (43.6%)^^** | **3.72 (63.6%)+**** |
| How difficult do you think it would be for this NICU to safely reduce its antibiotic use? | 111 | Neonatologists | 2.52 (1.85%) |  |
|  | 55 | Other prescribers | 2.20 (5.56%) |  |
| **Note**: The longitudinal cohort consists of prescribers who responded to both the pre- and post-OASCN survey (N=166). Other prescribers include neonatal fellows, hospitalists/pediatricians, neonatal nurse practitioners and physician assistants.  ^a^ Percent top-two box defined as the percent who answered “a little” or “very little” on the four-point Current Practice Likert items and “very” or “extremely” on the five-point Importance, Activity, and Capacity items  ^b^ Responses are in terms of inappropriate prescribing, so top-two box responses are “a little inappropriate prescribing” and “very little inappropriate prescribing  Pre-post differences: * p<0.05, ** p<0.01, *** p<0.001;  Pre-collaborative differences by type: ^ p<0.05, ^^ p<0.01, ^^^ p<0.001;  Post-collaborative differences by type: + p<0.05, ++ p<0.01, +++ p<0.001 | | | | |

**Table A.4:** Survey responses of longitudinal cohort, by NICU size (annual admissions volume)

| NICU Size | **Number of responses** | **NICU size category^a^** | **Pre-OASCN mean  (% top-two box^b^)** | **Post-OASCN mean  (%top-two box)** |
| --- | --- | --- | --- | --- |
| **Current practice** | | | | |
| In general, how would you rate the clinical appropriateness of antibiotic prescribing by clinicians in this NICU^c^? | 41 | Small | 3.14 (70.7%) | 2.97 (68.2%) |
|  | 47 | Medium | **3.00 (68.0%)** | **3.23 (76.6%)*** |
|  | 58 | Large | 3.36 (82.7%) | 3.43 (84.4%) |
| **Importance** | | | | |
| How important do you view antibiotic stewardship to be, compared to other priorities in this NICU? | 41 | Small | 4.22 (87.8%) | 4.36 (95.1%) |
|  | 48 | Medium | 4.16 (89.5%) | 4.18 (85.4%) |
|  | 59 | Large | 4.16 (79.6%) | 4.28 (86.4%) |
| In general, how important do you think **other clinicians** in this NICU view antibiotic stewardship to be, compared to other priorities in the NICU? | 41 | Small | **3.65 (73.1%)** | **3.90 (78.0%)*** |
|  | 48 | Medium | 3.81 (68.7%) | 3.95 (79.1%) |
|  | 58 | Large | 4.03 (75.8%) | 4.01 (81.0%) |
| How would you rate the importance placed on antibiotic stewardship by this **NICU’s leadership**? | 41 | Small | 4.24 (82.9%) | 4.36 (82.9%) |
|  | 48 | Medium | 4.27 (89.5%) | 4.39 (89.5%) |
|  | 59 | Large | 4.27 (81.3%) | 4.25 (91.5%) |
| How would you rate the importance placed on antibiotic stewardship by this **hospital’s leadership**? | 25 | Small | 3.96 (72.0%) | 4.24 (92.0%) |
|  | 40 | Medium | 4.00 (82.5%) | 3.02 (72.5%) |
|  | 40 | Large | 4.00 (80.0%) | 3.95 (72.5%) |
| **Activity** | | | | |
| How active has this **NICU** been in trying to improve antibiotic stewardship in the past year? | 38 | Small | **3.89 (68.4%)** | **4.34 (89.4%)*** |
|  | 45 | Medium | **3.91 (68.8%)** | **4.44 (93.3%)***** |
|  | 59 | Large | 3.93 (72.8%) | 4.16 (77.9%) |
| How active has this **hospital** been in trying to improve antibiotic stewardship in the past year? | 20 | Small | 3.85 (70.0%) | 4.00 (80.0%) |
|  | 38 | Medium | 3.78 (78.9%) | 4.00 (68.4%) |
|  | 39 | Large | 3.82 (71.7%) | 3.79 (69.2%) |
| **Capacity** | | | | |
| How would you rate the general openness of clinicians in this NICU to changing their antibiotic prescribing practice? | 41 | Small | 3.63 (48.7%) | 3.82 (68.2%) |
|  | 46 | Medium | 3.50 (54.3%) | 3.73 (58.7%) |
|  | 59 | Large | **3.83 (66.1%)** | **4.06 (79.6%)*** |
| How difficult do you think it would be for this NICU to safely reduce its antibiotic use? | 41 | Small | 2.19 (2.44%) |  |
|  | 46 | Medium | 2.32 (8.70%) |  |
|  | 58 | Large | 2.22 (3.45%) |  |
| **Note**: The longitudinal cohort consists of prescribers who responded to both the pre- and post-OASCN survey (N=166).  ^a^ NICU size based on total annual admissions volume, defined as small = ≤229 admissions, medium = >229 to ≤365 admissions, and large = >365 admissions  ^b^ Percent top-two box defined as the percent who answered “a little” or “very little” on the four-point Current Practice Likert items and “very” or “extremely” on the five-point Importance, Activity, and Capacity items  ^c^ Responses are in terms of inappropriate prescribing, so top-two box responses are “a little inappropriate prescribing” and “very little inappropriate prescribing  Pre-post differences: * p<0.05, ** p<0.01, *** p<0.001 | | | | |

**Table A.5:** Survey responses of all prescribers who completed pre- or post-OASCN survey

|  | **Pre-OASCN mean  (% top-two box^a^)**  **(N=256)** | **Post-OASCN mean  (% top-two box)**  **(N=194)** |
| --- | --- | --- |
| **Current Practice** |  |  |
| In general, how would you rate the clinical appropriateness of antibiotic prescribing by clinicians in this NICU^b^? | 3.26 (77.7%) | 3.23 (77.0%) |
| **Importance** |  |  |
| How important do you view antibiotic stewardship to be, compared to other priorities in this NICU? | 4.16 (84.7%) | 4.22 (88.6%) |
| In general, how important do you think **other clinicians** in this NICU view antibiotic stewardship to be, compared to other priorities in the NICU? | 3.85 (71.5%) | 3.92 (78.2%) |
| How would you rate the importance placed on antibiotic stewardship by this **NICU’s leadership**? | 4.15 (81.7%) | 4.30 (88.0%) |
| How would you rate the importance placed on antibiotic stewardship by this **hospital’s leadership**? | 3.70 (63.8%) | 3.90 (73.2%) |
| **Activity** |  |  |
| How active has this **NICU** been in trying to improve antibiotic stewardship in the past year? | 3.77 (68.4%) | 4.28 (86.3%) |
| How active has this **hospital** been in trying to improve antibiotic stewardship in the past year? | 3.54 (60.9%) | 3.90 (71.3%) |
| **Capacity** |  |  |
| How would you rate the general openness of clinicians in this NICU to changing their antibiotic prescribing practice? | 3.70 (61.9%) | 3.86 (68.7%) |
| How difficult do you think it would be for this NICU to safely reduce its antibiotic use? | 2.22 (5.16%) | - |

**Note**: Sample includes those who responses to both the pre-OASCN and post-OASCN survey

^a^ Percent top-two box defined as the percent who answered “a little” or “very little” on the four-point Current Practice Likert items and “very” or “extremely” on the five-point Importance, Activity, and Capacity items

^b^ Responses are in terms of inappropriate prescribing, so top-two box responses are “a little inappropriate prescribing” and “very little inappropriate prescribing
